# A Mixed-Method Approach to Develop and Validate an Integrated Food Literacy Tool for Personalized Food Literacy Guidance

**DOI:** 10.3389/fnut.2021.760493

**Published:** 2022-01-21

**Authors:** Tessy Boedt, Nele Steenackers, Joke Verbeke, Astrid Vermeulen, Charlotte De Backer, Peter Yiga, Christophe Matthys

**Affiliations:** ^1^Clinical and Experimental Endocrinology, Department of Chronic Diseases and Metabolism, KU Leuven, Leuven, Belgium; ^2^Laboratory of Molecular Bacteriology, Department of Microbiology, Immunology and Transplanation, Rega Institute, KU Leuven, Leuven, Belgium; ^3^Department of Communication Sciences, Faculty of Social Sciences, University of Antwerp, Antwerp, Belgium; ^4^Department of Food Technology, Kyambogo University, Kampala, Uganda; ^5^Department of Endocrinology, University Hospitals Leuven, Leuven, Belgium

**Keywords:** food literacy, personalized nutrition, validation, behavior change, eating, food, goals

## Abstract

**Background:**

Food literacy refers to all practicalities associated with healthy eating. Current food literacy tools are limited in practical use in clinical practice. Therefore, an integrated food literacy tool (IFLT) to assess food literacy and to personalize food literacy guidance was developed and validated.

**Methods:**

Following an iterative process, a food literacy framework was developed and food literacy goals were defined. A corresponding food literacy screener (FLS) to assess food literacy was developed along with an algorithm to provide personalized food literacy guidance based on the food literacy assessment. Content validation of the FLS was assessed by a panel of experts, measuring item and scale content validity index (I/S-CVI) and by the target population in semi structured interviews with 15 adults of reproductive age. Subsequently, an online cross-sectional survey was conducted among 114 adults of reproductive age to evaluate the validity of the FLS. Construct validity was examined against both the validated healthy eating and weight self-efficacy scale and against a food frequency questionnaire assessing healthy eating self-efficacy (HESE) and diet quality, respectively. Reliability was assessed with a two-week test-retest. Pearson correlation tests were conducted.

**Results:**

An IFLT consisting of a FLS and corresponding algorithm to personalize food literacy guidance by prioritizing food literacy goals was developed. The IFLT includes 24 food literacy goals, addressed by 17 FLS items. Every item received a weighting factor based on theory and expert opinion to prioritize food literacy goals according to personal needs. Content validity revealed that the FLS was rated relevant by experts (S-CVI = 0.93) and well-understood by the target population. The FLS has a good construct validity as it was positively correlated with diet quality (r = 0.536, *p* < 0.001) and with HESE (r = 0.685, *p* < 0.001). It also showed a good test-retest reliability (r = 0.721, *p* < 0.001).

**Conclusion:**

The newly developed IFLT is a practically applicable, context specific theory-and expert-based dual purpose tool to assess food literacy and to personalize food literacy guidance by prioritizing individuals' food literacy goals to their needs.

## Background

A healthy diet is essential for growth and development and can play a role in the prevention and management of many non-communicable diseases (1). Unfortunately, the diet of different (patient) populations is often far from optimal, even in those patient populations where a healthy diet is essential e.g., couples trying to conceive. A preconception healthy diet is not only beneficial for peoples' general health but also for their reproductive health and health of their offspring (2). One of the major challenges in optimizing diet is “how” to achieve a healthy diet. One potential way is by personalizing nutritional advice (3–5). The European Food4me study showed that personalized nutritional advice, via an internet-delivered intervention, produced larger and more appropriate changes in dietary behavior than a conventional approach among European adults (6, 7). Personalized nutrition (PN) is defined as “*individual-specific information founded in evidence-based science to promote dietary behavior change that may result in measurable health benefits*.”(3). Moreover, personalization constitutes an effective behavior change method according to the intervention mapping protocol (8). Next to the question of “how” to achieve a healthy diet, there is the question of “what (i.e., the content of nutritional advice)” needs to be optimized. Achieving and maintaining a healthy diet comprises more than just an optimal combination of food items. More specifically, knowledge, skills and individual behavior should be taken into account when aiming to optimize nutritional health. Evidence depicts food literacy as an effective strategy to counter these dietary behavior determinants (9, 10).

Food literacy acknowledges multiple determinants including knowledge, skills and self-efficacy on various practicalities associated with healthy eating such as food planning, selecting food items, food preparation, eating and evaluating information about food. Multiple research groups defined food literacy with the most cited model created by Vidgen and Gallegos (11). To define food literacy, Vidgen and Gallegos combined the perspectives of food experts and of urban disadvantaged young people (11). However, the application to other populations is currently unknown. To broaden validity and acceptability, a review identified six similar themes across existing food literacy models including (i) skills and behavior; (ii) food/health choices; (iii) culture; (iv) knowledge; (v) emotions and (vi) food systems. The review highlighted that most definitions focus on critical knowledge (e.g., information and understanding), while only a few definitions incorporate functional knowledge (e.g., skills, abilities and choices) (10).

Next to heterogeneity in food literacy models, a plethora of tools have been developed for measuring food literacy. However, their practical use in clinical practice remains limited and they were all developed for a specific context (12–16). A recent review on food literacy tools in adults found five tools to measure (parts) of food literacy, one on food literacy strategy indicators, three to evaluate a specific food literacy intervention and three to measure food literacy as a characteristic within a broader study (12). The majority of these tools (7/12) followed the model of Vidgen and Gallegos but there was large variation (i) how the definition was applied and (ii) the specific components of food literacy that were assessed. For example, a Swiss research group developed and validated a short food literacy questionnaire (SFLQ) based on Swiss dietary recommendations and Nutbeam's model of critical, interactive and functional health literacy to evaluate a food literacy intervention on salt reduction in the workplace (15, 17). This questionnaire focuses on people's understanding and search capacities of information about a healthy diet and their ability to judge and use this information. Poelman et al. developed a more general self-perceived food literacy scale (SPFL) with respect to healthy eating among Dutch adults. They followed the model of Vidgen and Gallegos as well as aspects of health literacy as described by Vidgen and Gallegos (11) and Sorensen et al. (18, 19). To the best of our knowledge, none of the existing tools were content-validated within the target population, which is important as food literacy is an everyday practice and should reflect people's lived experience (12).

As food literacy is complex, highly context- and culture-dependent, food literacy tools should be developed and validated in the appropriate context (20). In addition, the challenge remains how to personalize nutritional advice using the concept of food literacy. Therefore, the aim of this study was to develop and validate an integrated food literacy tool with a dual purpose: (i) assessing food literacy and (ii) personalizing food literacy guidance in adults of reproductive age.

## Methods

**Figure 1:**
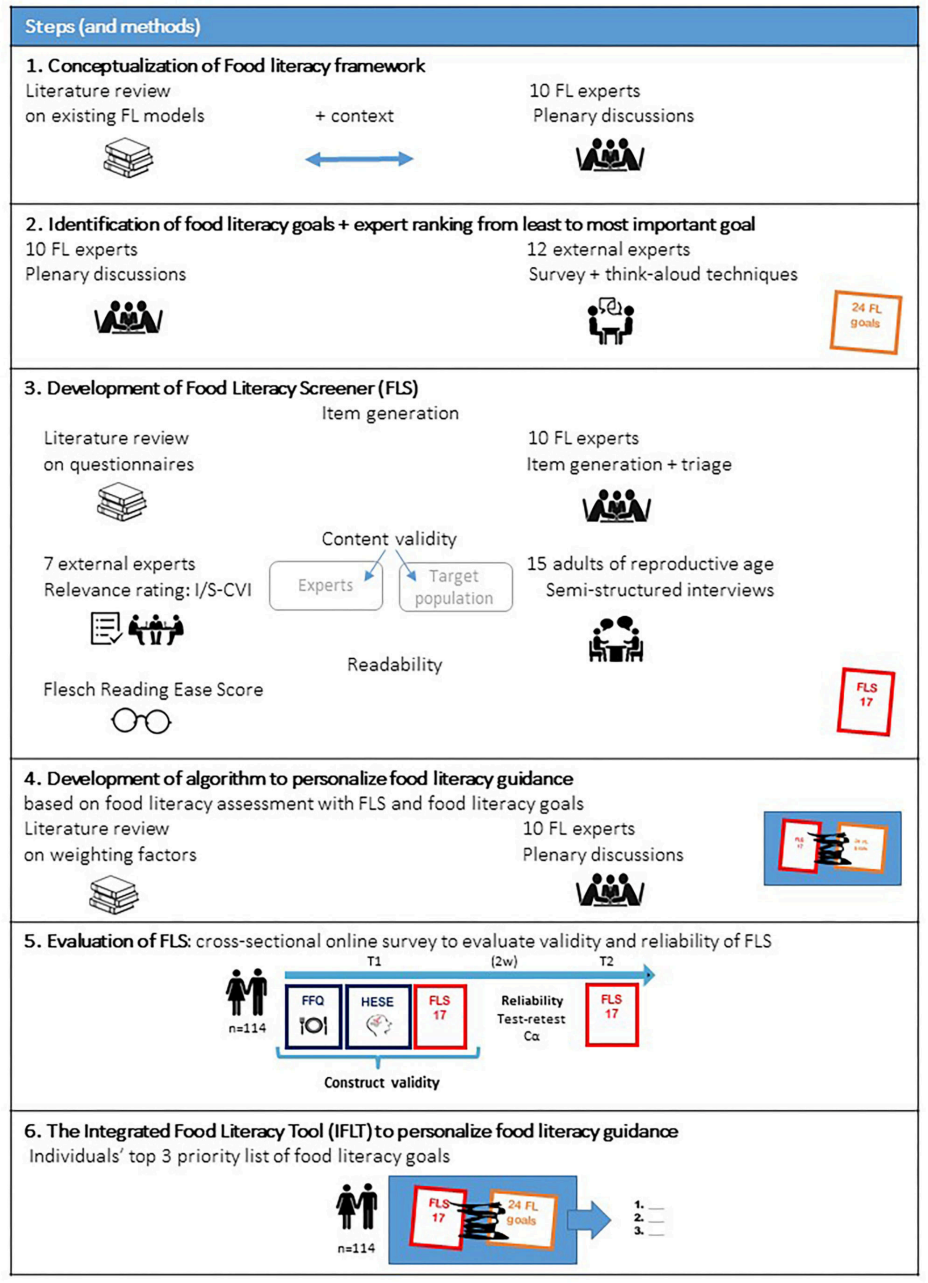
Step-wise approach for IFLT development. FL, Food Literacy; I-CVI, Item Content Validity Index; S-CVI, Scale Content Validity Index; FLS, Food Literacy Screener; IFLT, Integrated Food Literacy Tool; FFQ, Food Frequency Questionnaire; Cα, Cronbach's alpha; HESE, Healthy Eating Self-Efficacy.

Previously, our group developed a randomized controlled trial to assess the effects of a mobile preconception lifestyle program in couples of reproductive age (21, 22). The program included a personalized food literacy intervention to promote a healthy diet. To personalize the food literacy component, an integrated food literacy tool (IFLT) was developed consisting of a food literacy screener (FLS) and an algorithm to prioritize food literacy goals according to personal needs based on the assessment with the FLS. To develop and evaluate the IFLT, a step-wise mixed method approach was applied based on scale development methods by Polit and Beck (23), Patrick et al. (24, 25) and on the STROBE-nut guidelines (Strengthening The Reporting of OBservational Studies in Epidemiology – Nutritional epidemiology) (26). Figure 1 presents an overview of the step-wise approach for the IFLT development. This study was approved by the Social and Social Ethics Committee of KU Leuven (G2018101360).

### Conceptualization of Food Literacy Framework

A literature review regarding existing food literacy models was conducted. Subsequently, the identified models were compared and discussed with a team of 10 Belgian experts (two professors and eight Phd students from two Belgian Universities) working in the field of nutrition and food literacy (FL experts), taking Belgian food culture, context and guidelines into account (27, 28). First, determinants and domains of food literacy were addressed, followed by a discussion on a definition of food literacy. Multiple plenary discussions were held until consensus regarding a context-specific food literacy framework in the Belgian culture was reached.

### Identification of Food Literacy Goals

Because goalsetting was identified as an important behavior change technique (21), the FL experts were asked to translate the food literacy framework into practical and applicable food literacy goals. To guide the food literacy goal identification, a matrix was presented to the FL experts that combined the determinants and domains of food literacy. A final set of goals was obtained after an iterative process of consulting the FL experts.

According to the principles of the think-aloud methodology (29), 12 other external independent experts in the field of nutrition (dieticians working in University Hospitals Leuven) were asked to identify any missing and otiose food literacy goals and rate them on a seven-point Likert scale from 1: “Not relevant at all” to 7: “Extremely relevant.” Based on this input, several food literacy goals were defined and ranked from least important to most important goal according to the external expert relevance rating.

### Development of Food Literacy Screener

#### Item Generation

Based on the literature review, existing questionnaires on food literacy and existing questionnaires covering different determinants of food literacy, items for a FLS were generated by two of the 10 FL experts (TB and AV). After creating a comprehensive list, the remaining eight FL experts triaged the items in multiple plenary sessions until consensus was reached. The triage aimed to reduce the number of items, while meeting the condition that every item should reflect at least one food literacy goal. Items were included if they were relevant to the defined food literacy concept and goals and if they were appropriate for the target population.

#### Content Validity and Readability Evaluation

To ensure expert content validity, another independent panel of nutrition experts (*n* = 7, including one professor, two independent dieticians and four PhD students working in the field of nutrition) who had not been involved in the previous development steps was recruited. These external experts were asked to evaluate every question on a four-point Likert scale from 1: “Not relevant” to 4: “Very relevant to the concept of food literacy” to calculate an item and scale content validity index (I-CVI & S-CVI). To be relevant, the I-CVI and S-CVI needed to be at least 0.78 and 0.9, respectively (23). I-CVI was calculated by dividing the number of experts scoring three or four by the total number of experts. S-CVI is the average of all I-CVIs. Next, content validity or respondent understanding was assessed within the target population. A total of 15 participants of reproductive age were invited to participate in a semi-structured cognitive interview in order to evaluate the understandability of the FLS. Participants were recruited through social media and snowball sampling. A study team member provided information to participants and encouraged them to pass it on to others who may be interested or eligible. During the interview, participants were encouraged to complete our FLS while explaining out loud how they interpreted every item. All interviews were recorded and qualitative analysis was performed according to the principles of Patrick et al. (24). Clarity and reading level of our FLS was evaluated by calculating the Flesch reading ease score using the following formula = 206.835 – 1.015 × (Total Words / Total Sentences) – 84.6 × (Total Syllables / Total Words). Higher scores indicate material that is easier to read. A score between 70 and 80 is desirable as it reflects that the questionnaire is fairly easy to read for the average adult (23).

### Development of the Algorithm to Personalize Food Literacy Guidance

Next to the FLS in which every item reflects at least one food literacy goal to assess an individual's food literacy, an algorithm was developed to provide personalized food literacy guidance based on the food literacy assessment. The external expert ranking of the food literacy goals (step 2) was combined with literature review and FL expert opinion to prioritize the food literacy goals to individuals' needs by linking weighted factors to each answer option of the FLS.

### Evaluation of Food Literacy Screener

#### Study Design and Setting

To evaluate the validity and reliability of the FLS, a cross-sectional online survey was conducted among adults of reproductive age from January to April 2019 with an online survey platform, Qualtrics© (Qualtrics, Provo, UT) (30). The study design is visualized in Figure 1, step 5.

#### Participants

Belgian Dutch-speaking volunteers of reproductive age (18–43 years old) were recruited through social media and snowball sampling to complete the online survey. A study team member provided information to participants and encouraged them to pass it on to others who may be interested or eligible. Responses of participants with specific dietary requirements due to pregnancy, diabetes mellitus, coeliac diseases, intolerances or malabsorption were excluded. We strived to recruit at least 100 participants, a reasonable sample size for validation studies according to Willett (31).

#### Measures

After receiving written informed consent, we collected the data on sociodemographics, food literacy, food intake, diet quality and healthy eating self-efficacy: (**i**) Sociodemographic data included age, gender, ethnicity, child wish, self-reported health, and education level. (ii) Food literacy was assessed using our newly developed 17-item FLS (step 3). To achieve an overall food literacy score, all items were recoded to a 7-point nominal scale (IOS) and summated. This overall food literacy score was recalculated to a score of 100, where, a score of 0 suggests poor food literacy and a score of 100 suggests excellent food literacy. (iii) Food intake and diet quality were assessed using the validated Belgian Food Frequency Questionnaire (FFQ) (32). The FFQ questions frequency and portion size of consumption of foods and beverages. An overall diet quality index was calculated from the FFQ according to Huybrechts et al. (33). The diet quality index (ranging from 0 to 100) reflects compliance with Belgian food-based dietary guidelines taking into account dietary quality, dietary diversity dietary equilibrium. The higher the score, the better the diet quality. (iv) In addition, healthy eating self-efficacy (HESE) was assessed using the seven-item healthy eating self-efficacy subscale of the Healthy Eating and Weight Self-Efficacy scale (HEWSE) (34). The higher the score, the better the healthy eating self-efficacy.

#### Statistical Methods and Analysis

Analyses were performed using SPSS Statistical Software (35). As there is no golden standard to validate food literacy tools, we evaluated the construct validity of the FLS against dietary variables that were expected to correlate with food literacy, including diet quality and healthy eating self-efficacy. Pearson correlation tests were conducted. Good construct validity is indicated by correlations below 0.9, but above 0.4 (23). To assess reliability, participants had to fill in the FLS twice with a two-week interval. Interclass correlation coefficients were calculated. The cutoff value for reliability was 0.7 (23). Additionally, internal consistency was assessed by calculating Cronbach's alpha. A value of Cronbach's alpha above 0.7 was considered to be an indicator of adequate reliability (23). To evaluate concurrent validity, overall food literacy scores were compared between men and women and between people with or without a child wish using independent *t*-tests (*p* < 0.05 was used to determine statistical significance). Pearson correlations were used to assess if there was a relation between the total food literacy score and age, self-reported health and education level.

### The Integrated Food Literacy Tool: Prioritizing Individuals' Food Literacy Goals to Personalize Food Literacy Guidance

For every participant of the cross-sectional survey, the newly developed algorithm (step 4) was applied to determine the priority list of food literacy goals. The IFLT prioritized the food literacy goals from 1 to 24 based on participants' answers to the FLS and the algorithm. For the “eat”-related food literacy goals, we evaluated the accuracy (i.e., the ability to personalize food literacy guidance to individual needs) by comparing with data from the FFQ. If participants received a food literacy goal on eating in their top three priority list, their actual food intake from the FFQ was evaluated and corrected for energy intake. To assess if participants were correctly assigned relevant food literacy goals, this relative food intake was compared to the Belgian food-based dietary recommendations. IFLT accuracy was calculated as the number of persons with a food literacy goal on eating that were correctly classified (i.e., in top three priority list and not fulfilling the Belgian food-based dietary recommendations according to the FFQ for that goal) divided by the total number of participants with that food literacy goal on eating in their top three priority list.

## Results

### Conceptualization of Food Literacy Framework

The conceptualization of a food literacy model adapted to the Belgian culture and context was based on practical food literacy definitions and models with structured domains, such as the model of Vidgen and Gallegos describing four domains (plan, select, prepare, and eat) and the model of Perry et al., who define five attributes of food literacy (food skills, knowledge, self-efficacy, and ecologic and food decisions) (11, 20, 36). Regarding the different determinants on food literacy, the FL expert panel agreed to implement knowledge, skills and self-efficacy. Regarding domains on food literacy, the FL expert panel came to a consensus to focus on planning, selecting, preparing, eating and evaluating information. After four plenary sessions, the FL expert panel agreed to the following food literacy definition: “*Food literacy is the interrelated combination of knowledge, skills and self-efficacy on food planning, selecting foods, and food preparation, eating and evaluating information about food with the ultimate goal of developing a lifelong healthy, sustainable and gastronomic relationship with food*.” The first domain of food planning focusses on the ability to schedule and to make time to prepare healthy meals and to make healthy food choices. It also reflects on the ability to access healthy foods in various contexts such as at work, when having little time or when eating out. Secondly, the domain of selecting foods consists of the knowledge and skills, which allows to discriminate a variety of foods and to evaluate their effects on individual and collective well-being. This domain is followed by food preparation, which relates to the functional competences that are required to prepare available foods and to apply the principles of safe food hygiene. In the domain of eating, the focus lies on enhancing the consumption of plant-based and fiber-rich food items and to reduce the intake of processed foods such as processed meat and snacks according to the Belgian FBDG. Furthermore, a better understanding of the consequences of food-related decisions on individual well-being (in the short and long term) is an important aim of this domain. Lastly, the domain of evaluating information was added (37). This incorporates the ability to access, interpret and use nutrition information.

### Identification of Food Literacy Goals

The FL expert panel translated the Belgian food literacy model into 22 practical and applicable food literacy goals. Of these, external experts considered the goal of knowledge of FBDGs superfluous. Moreover, they suggested combining goals on variation in foods and highlighted the need to add a goal on eating in stressful situations. These suggestions led to 24 goals on food literacy. Based on the external expert relevance rating, these 24 food literacy goals were ranked from 0.01 (least important goal) to 0.24 (most important goal). Table 1 presents an overview of the food literacy goals suggested by the 10 FL experts and 12 external experts, the ranking of the goals by the 12 external experts (the higher the score the higher the relevance) and the corresponding 17 items of the FLS.

**Table 1:** Overview of the food literacy goals, expert ranking, corresponding items of the FLS and I/S-CVI.

**Food literacy goals (*n* = 24)**	**Expert ranking: the higher, the more relevant**	**Food literacy screener (FLS) (*n* = 17)**	**Scoring: 7-point Likert scale + situations + frequency of advised portions**	**Item content validity index (I-CVI)**
**Plan**				
G1: Making time to eat (together)	0.21	Q1: How often do you make time to eat?	Always/never	1
Always having access to healthy food		Q2: In which situation do you experience the most difficulties to follow a healthy diet?	Situation	1
•G2: When you have little time	0.06		•When you have little time	
•G3: At home	0.16		•At home	
•G4: At work/school/on the road	0.09		•At work/school/on the road	
•G5: When eating out	0.02		•When eating out	
•G6: When stressed	0.03		•When stressed	
**Select**				
G7: Making healthy food choices	0.24	Q3: I can choose the right food items in order to achieve a healthy diet	Agree/disagree	1
G8: Understanding food packages and labels	0.8	Q4: I understand what's on food packages	Agree/disagree	0.86
G9: Variation (in selecting preparing and eating)	0.15	Q5: I vary my food choices	Agree/disagree	1
**Prepare**				
G10: Being able to compose a healthy meal	0.20	Q6: I can compose a healthy meal	Agree/disagree	1
G11: Being able to know and apply basic cooking skills	0.04	Q7: I can cook	Agree/disagree	0.86
G12: Knowing and applying principles of food hygiene	0.01	Q8: I can apply the principles of food hygiene	Agree/disagree	0.71
**Eat**				
G13: Understanding benefits of healthy eating	0.14	Q9: What I eat influences my health	Agree/disagree	0.86
G14: Eating more plant-based (less animal-based)	0.05	Q10: How often do you eat a portion of meat?	Frequency of advised portion	1
G15: Eating enough vegetables	0.23	Q11: How often do you eat a portion of vegetables?	Frequency of advised portion	1
G16: Eating enough fruits	0.11	Q12: How often do you eat a portion of fruit?	Frequency of advised portion	1
G17: Drinking enough and mainly water	0.22	Q13: How much water do you drink per day?	Frequency of advised portion	1
G18: Eating less ultra-processed foods	0.10	Q14: How often do you eat savory and/or sweet snacks?	Frequency of advised portion	1
G19: Eating consciously and not too much	0.13	Q15: How often do you eat too much	Always/never	0.86
Eating healthy at		Q16: During which meal do you experience the most difficulties to follow a healthy diet?	Situatio	1
•G20: Breakfast	0.17		•Breakfast	
•G21: Lunch	0.19		•Lunch	
•G22: Diner	0.18		•Diner	
•G23: Snacking	0.12		•Snacking	
**Information**				
G24: Being able to find reliable information about a healthy diet	0.07	Q17: If I have questions regarding a healthy diet, I can find reliable information on this	Agree/disagree	0.71
**Scale Content Validity Index (S-CVI)**				**0.93**

*The bold value indicates Scale Content Validity Index (S-CVI)*.

### Development of Food Literacy Screener

Based on existing nutrition knowledge questionnaires, self-efficacy questionnaires, dietary behavior questionnaires and additional item generation, two FL experts created a first comprehensive list of 60 items (15, 31, 38, 39). Together with the other 8 FL experts, this list was reduced in four rounds of internal review by excluding duplicate items, items that questioned the same goal and items that did not meet our food literacy goals. This resulted in a 17-item food literacy screener (FLS), with every item reflecting at least one goal (Table 1).

The content validity assessment with 7 independent nutrition experts revealed good relevance of our FLS (S-CVI = 0.93). Two items were rated as less relevant (I-CVI = 0.71) including item 17 on information and item eight on hygiene. An overview of the I-CVI is presented in Table 1.

The content validity assessment with the target population revealed that our FLS was clear and well-understood by adults of reproductive age, that no items were missing and that the length of the FLS was acceptable. However, some participants remarked that in the items on portion size, the difference between two answering possibilities was too large. Therefore, we rephrased the options for these questions. After adjustments, the Flesch reading ease score of the FLS was 75.6, reflecting that our FLS was fairly easy to read for the average adult.

### Development of the Algorithm to Personalize Food Literacy Guidance

The algorithm to personalize food literacy guidance included the following requirements: (1) Every item of the FLS was allocated to one or more food literacy goals (Table 1); (2) Every answer option of every FLS item received an item priority score (IPS) allowing to prioritize food literacy goals to individual needs. For example, a higher IPS was provided, if people would benefited from focusing on the respective food literacy goal (Table 2); (3) A default prioritization order of food literacy goals was generated with the food literacy goals receiving a score from 0.01 to 0.24 (expert ranking weight) (Table 1). The algorithm was applied as follows: after completion of the FLS, the IPS for every food literacy goal was generated and summed with the default prioritization score (expert ranking weight). For example, if participants had a perfect food literacy they would receive the 24 goals in the default prioritization order. This algorithm allows to prioritize food literacy goals from 1 to 24 based on individual FLS answers. Table 2 presents an example of the scoring and prioritizing process for the food literacy items and respective goals: “Making healthy food choices” and “Drinking enough and mainly water.” The IFLT operates the algorithm and consequently creates different tips in line with the selected food literacy goals.

**Table 2:** Example of scoring and prioritizing process.

**Goal behind item**	**Expert ranking weight**	**Item to address goal**	**Item score for calculating overall food literacy (IOS)**	**Item score for prioritizing food literacy goals to individual needs (IPS)**
Making healthy food choices	0.24 (Most important goal = highest weight)	**I can choose the right food items in order to achieve a healthy diet**		
		Strongly disagree	0	5 (would benefit from
		Disagree	1	4 focusing on this goal)
		Partly disagree	2	3
		Not agreeing, not disagreeing	3	2
		Partly agree	4	1
		Agree	5	0 (no individual needs to
		Strongly agree	6	0 focus on this goal)
Drinking enough and mainly water	0.22	**How much water do you drink per day? (1 glass** **=** **200 ml)**		
		Less than 1 glass per day	0	5 (would benefit from
		1–3 glasses per day	1	4 focusing on this goal)
		3–5 glasses per day	2	3
		5–7 glasses per day	3	2
		7–8 glasses per day	4	1
		8–10 glasses per day	6 (recommended =	0 (no individual needs to
		More than 10 glasses per day	5 highest score)	0 focus on this goal)

### Evaluation of Food Literacy Screener

#### Participants

In total, 114 participants completed the online survey. Participants were 28 (SD: ± 5.36) years old on average and were all Caucasian. The majority of participants scored their health as good or very good (75%) and were highly educated (90%). Approximately half of the participants were women and one-third had an active child wish. Table 3 presents an overview of participants' characteristics.

**Table 3:** Participants' characteristics.

**Participants' characteristics**	**Participants in content validity FLS (*****n*** **=** **15)**		**Participants in overall evaluation FLS (*****n*** **=** **114)**	
	**Count**	**%**	**Count**	**%**
**Sex**				
Women	9	60%	62	54%
Men	6	40%	52	46%
**Childwish**				
Yes	5	33%	39	34%
No	10	67%	75	66%
**Self-related health**				
Very bad	0	0%	0	0%
Bad	0	0%	3	3%
Not bad / Not good	3	20%	26	23%
Good	8	53%	77	68%
Very good	4	27%	8	7%
**Education level**				
No degree	0	0%	1	1%
High school degree	2	13%	10	9%
Bachelor degree	8	53%	45	39%
Master degree	5	33%	56	49%
PhD	0	0%	2	2%

#### Validity and Reliability of the Food Literacy Screener

**Figure 2:**
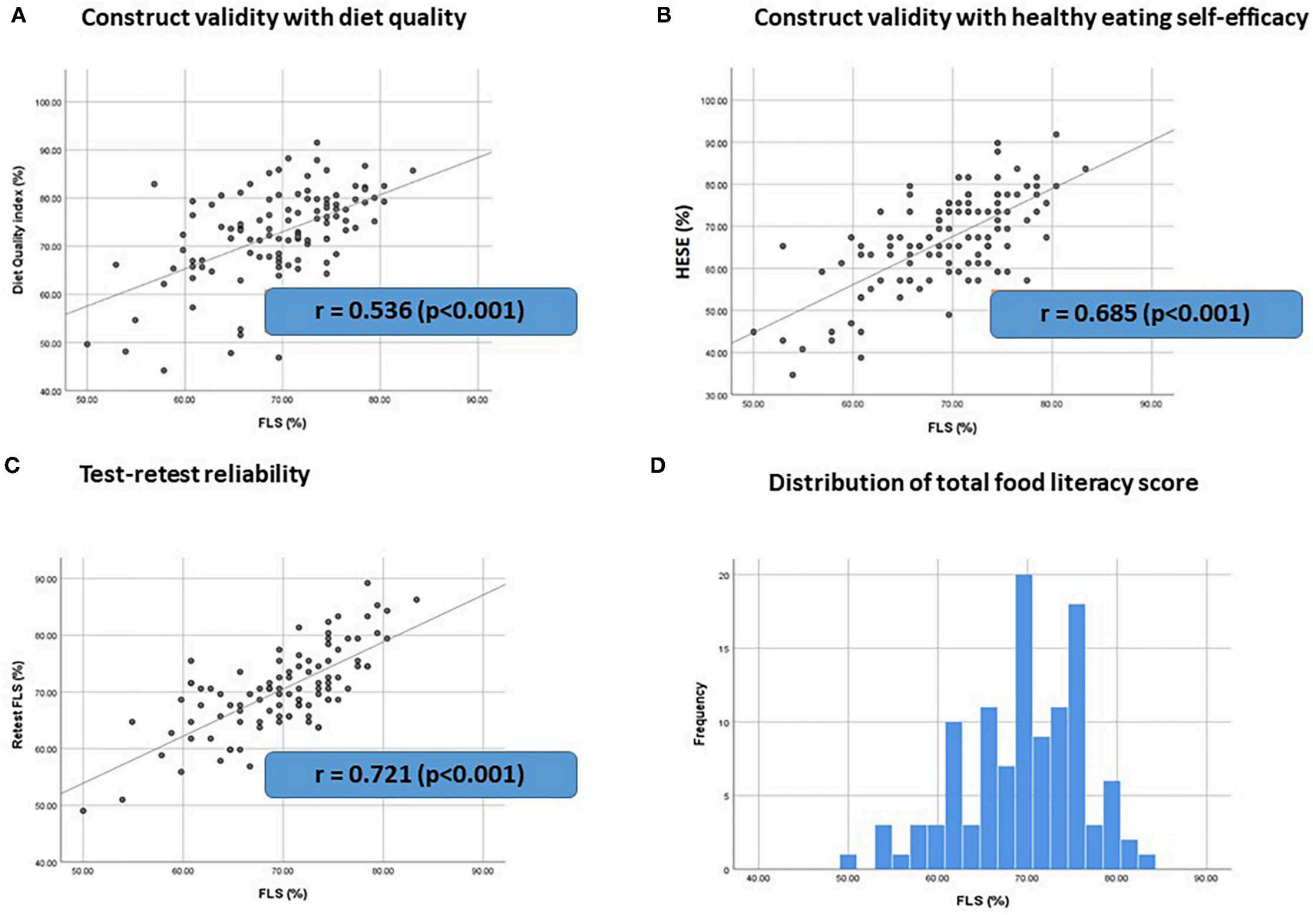
FLS Validity and reliability. (*n* = 114) **(A,B)** Construct validity of the FLS against dietary variables **(A)** diet quality and **(B)** healthy eating self-efficacy (HESE) that are expected to be related with food literacy. r = Pearson correlation coefficient. **(C)** Test-retest reliability of the FLS. r = interclass correlation coefficient. **(D)** Distribution of total food literacy score.

Figure 2 presents an overview of FLS validity and reliability.

Regarding construct validity, correlation analyses indicated that food literacy was positively correlated with diet quality (r = 0.536, *p* < 0.001) and with HESE (r = 0.685, *p* < 0.001) as presented in Figure 2. These findings confirm good construct validity (r > 0.4) of the FLS against dietary variables that are expected to be related with food literacy.

Regarding reliability, test-retest indicated good reliability of the FLS (r = 0.721, *p* < 0.001). The internal consistency of the overall scale was not sufficient (Cronbach's alpha = 0.558).

Regarding concurrent validity, the average total food literacy score was 69% ± 6.7 for our participants and ranged from 50 to 83%. There were no significant differences in the total food literacy score of men (69% ± 7.0) and women (70% ± 6.4) (*p* = 0.565) and between people with (70% ± 6.6) or without a child wish (69% ± 6.8) (*p* = 0.507). Additionally, correlation analyses revealed no correlation between the total food literacy score and age (r = 0.127, *p* = 0.142) or education level (r = 0.147, *p* = 0.122). Self-reported health was positively but weak correlated with food literacy (r = 0.367, *p* < 0.001) (see Supplementary Figure 1).

### The Integrated Food Literacy Tool: Prioritizing Individuals' Food Literacy Goals to Personalize Food Literacy Guidance

**Figure 3:**
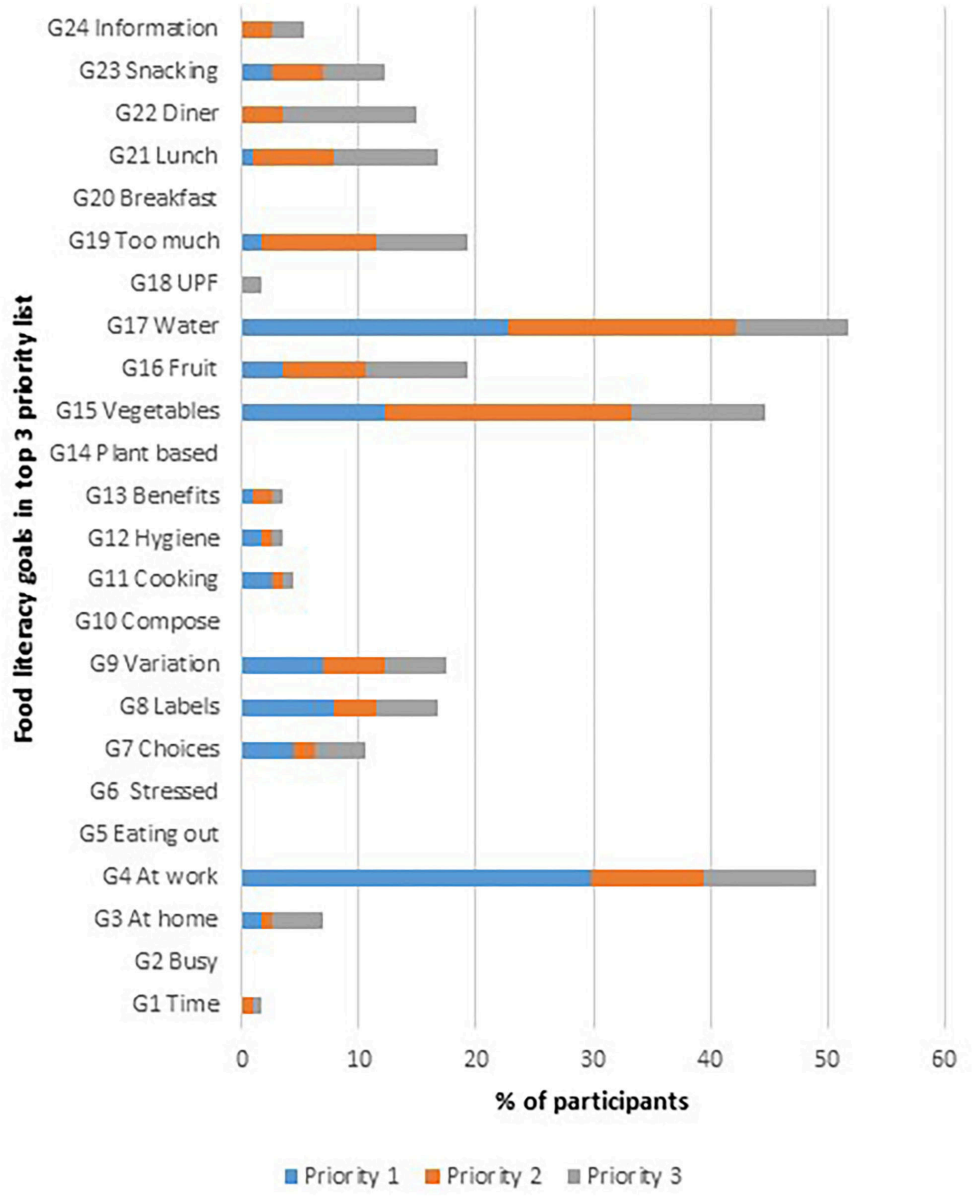
Top 3 Priority list of food literacy goals calculated with the IFLT (*n* = 114).

Figure 3 presents an overview of the top 3 priority lists of food literacy goals for the 114 participants. The goals that appeared most in participants' top 3 priority lists were: goal 4 “*Having access to healthy food at work/school/on the road*” in 50 % of participants, goal 15 “*Eating enough vegetables*” in 44 % of participants and goal 17 “*Drinking enough and mainly water*” in 52% of participants. Goal 2 “*Having access to healthy food when you have little time*”, goal 5 “*Having access to healthy food when eating out*”, goal 6 “*Having access to healthy food when stressed*”, goal 10 “*Being able to compose a healthy meal*”, goal 14 “*Eating more plant-based*”, and goal 20 “*Eating healthy at breakfast*” were absent from all participants' top 3 priority lists. The goals considered as the lowest priorities were: goal 5 “*Having access to healthy food when eating out*” in 39% of participants and goal 12 “*Knowing and applying principles of food hygiene*” in 60 % of participants. These findings are in line with the experts' ranking, who rated these goals as well as the least important goals.

Table 4 presents the accuracy of the IFLT. Evaluating if participants with one of the five food literacy goals on eating in their top 3 priority list were actually in need of working on that goal revealed an accuracy between 88 and 100%.

**Table 4:** Accuracy of correctly assigning food literacy goals on eating to participants' needs.

**Goals on eating**	**Eating more plant-based**		**Eating enough vegetables**		**Eating enough fruits**		**Drinking enough and mainly water**		**Eating less ultra-processed foods**	
*n* = 114	FFQ meat intake >100 g/day + top 3	top 3	FFQ vegetable intake <300 g/day + top 3	top 3	FFQ fruit intake <250 g/day + top 3	top 3	FFQ water intake <1,500 ml/day + top 3	top 3	FFQ ultra-processed food intake >50 g/day + top 3	top 3
n	0	0	50	51	20	22	52	59	2	2
% Accuracy	100%		98%		91%		88%		100%	

## Discussion

To the best of our knowledge, this is the first study that developed and evaluated a practically applicable, context- specific, theory- and expert-based dual purpose tool to assess food literacy on the one hand and to create personalized food literacy guidance by prioritizing food literacy goals to individual needs on the other hand. The IFLT consists of a 17-item FLS and an algorithm to prioritize 24 food literacy goals according to individual needs. This tool was rated relevant by experts and well-understood by the target population. The context or culture dependence of food literacy was visible in the food literacy goals priority lists of the participants and in the relevance ranking by the experts. For example, the goals that appeared most in the top 3 priority lists of the participants are in accordance with the current dietary behavior of the Belgian population (i.e., low vegetable and water intake) (40). The goal on having access to healthy food when at work, at school or on the road also frequently featured in the top 3 priority list of food literacy goals of this population, emphasizing the busy life stage of adults of reproductive age and potential lack of healthy food environment (41). The goals on food hygiene, and on having access to healthy food when eating out were considered as the lowest priority in most participants and were also ranked as least relevant by experts. The Belgian population lives in a culture where food hygiene is self-evident. Belgians perceive themselves as a population that likes to go out for a lavish meal. These findings once more highlight the importance of context and culturally adapted food literacy tools, and development and evaluation within the target population (20).

Similar to other existing food literacy tools as discussed in the introduction, our FLS showed good construct validity and test re-test reliability. However, our internal consistency calculated with Cronbach's alpha was low, which is caused by the multidimensionality and dual purpose of our tool. Food literacy consists of a wide variety of features and comprehensibility and context dependence makes short quantitative assessment of food literacy challenging. We acknowledge that a more comprehensive questionnaire following detailed approaches on scale development and validation including factor analyses such as recently described by Boateng et al. would add scientific value. However, the purpose of our study was to develop a short food literacy screener able to personalize food literacy guidance *and not to perform a “classic” validation of a questionnaire* (42). Future research could use existing and newly developed food literacy tools synergistically to obtain a better understanding of the concept of food literacy. Additionally, future research should also focus on investigating measures beyond the individual scope of food literacy, including environmental and societal factors such as sustainability. This was not covered in our tools as we focused on the individual scope of food literacy.

Other limitations of our study are the potential lack of a representative sample. Our sample of experts included mostly women working in two university hospitals. Our sample of the target population included people with a higher education level than that of the average Belgian population of reproductive age, this might be selection bias due to the snowball sampling. Furthermore, cut-off values of the FLS score to categorize participants into groups with bad, good or excellent food literacy are currently not available. Future research could include capacity to distinguish evaluation with dieticians or other populations and use this or other (food literacy) tools in different populations and clinical settings to create insights in food literacy of the broader population and expand the external validity.

Another limitation was that our measures in the cross-sectional survey were self-reported. This may have caused bias in that participants expressed their ideas rather than their actual behavior or induced a social desirability bias. However, these are common limitations in the field of behavioral nutrition research (43). The implementation of objective markers such as anthropometric data could contribute to monitor this shortcoming. In addition, collecting anthropometric data of the participants could be of great value to evaluate a link between food literacy and diet-related diseases such as obesity.

Regarding the prioritization of food literacy goals, we were only able to evaluate the accuracy of food literacy goals on eating and not evaluate if all food literacy goals were correctly assigned to the needs of participants. Future studies should include or develop measurement tools that can be used to objectively evaluate correct assignment of the other food literacy components including planning, selecting, preparing and finding information about food.

To our knowledge, this is the first tool to create personalized guidance on food literacy. Other current studies using PN focused primarily on dietary intake. For example, in the Food4Me study, dietary intake was assessed and personalized using an online food frequency questionnaire (5, 6). Also in a recent Japanese pilot study the primarily focus was on dietary intake (44). Maintaining a healthy diet comprises more than just eating an optimal combination of food items. Focusing on food literacy can be an effective strategy to optimize nutritional health (9, 10).

The strengths of this study include: (i) the mixed-method approach; (ii) theory- and expert-based context and culturally dependent tool development; (iii) content validity assessment with experts as well as with the target population; (iv) the practical applicability to use this tool to assess food literacy and to personalize food literacy guidance, with the aim to improve food literacy and dietary behavior. Our study showed a positive relation between food literacy and diet quality. In the PreLiFe-RCT we will evaluate if a personalized food literacy intervention using the IFLT will also improve the diet quality and dietary behavior of people trying to conceive (21). Future effectiveness studies in other populations and clinical settings should further explore if (personalized) food literacy guidance can improve the dietary behavior and related clinical markers.

## Conclusion

In conclusion, we developed and evaluated the first dual purpose tool to assess food literacy on the one hand and to create personalized food literacy guidance on the other hand. This integrated food literacy tool consists of a 17-item food literacy screener to assess food literacy and an accompanying algorithm to prioritize 24 food literacy goals according to individual needs. Future studies in other populations and clinical settings should further explore the effectiveness of our tool and evaluate if (personalized) food literacy guidance can improve the dietary behavior and related clinical markers. If so, national and international public health nutrition programs could incorporate this broad range of practicalities that comprise food literacy into their approach to improve the dietary behavior of their population.

## Data Availability Statement

The raw data supporting the conclusions of this article will be made available by the authors, without undue reservation.

## Ethics Statement

The studies involving human participants were reviewed and approved by the Social and Social Ethics Committee of KU Leuven (G2018101360). The patients/participants provided their written informed consent to participate in this study.

## Author Contributions

TB and AV made an essential effort for the acquisition of the patient data. TB analyzed. TB, NS, AV, JV, PY, CB, and CM interpreted the data. TB and CM prepared this manuscript. All authors contributed substantially to the development of the integrated food literacy tool, read, revised, and approved the final manuscript.

## Funding

This work was supported by the Research foundation Flanders (Belgium) (FWO-TBM; Reference: T005417N).

## Conflict of Interest

The authors declare that the research was conducted in the absence of any commercial or financial relationships that could be construed as a potential conflict of interest.

## Publisher's Note

All claims expressed in this article are solely those of the authors and do not necessarily represent those of their affiliated organizations, or those of the publisher, the editors and the reviewers. Any product that may be evaluated in this article, or claim that may be made by its manufacturer, is not guaranteed or endorsed by the publisher.

## Abbreviations

Cα: Cronbach's alpha
IOS: Item score for calculating overall food literacy
IPS: Item score for prioritizing food literacy goals to individuals&#8217 needs
FFQ: Food Frequency Questionnaire
FL: Food Literacy
FLS: Food Literacy Screener
HEWSE: Healthy Eating and Weight Self-Efficacy
HESE: Healthy Eating Self-Efficacy
IFLT: Integrated Food Literacy Tool
I-CVI: Item Content Validity Index
PN: Personalized Nutrition
S-CVI: Scale Content Validity Index
STROBE-nut: Strengthening the reporting of observational studies in epidemiology &#8211 Nutritional epidemiology
UPF: Ultra-Processed Foods.

## References

[1] WHO. Global Action Plan for the Prevention and Control of Noncommunicable Diseases 2013–2020. Geneva; World Health Organisation (2013).

[2] Stephenson J Heslehurst N Hall J Schoenaker D Hutchinson J Cade JE. Before the beginning: nutrition and lifestyle in the preconception period and its importance for future health. Lancet. (2018) 391:1830–41. 10.1016/S0140-6736(18)30311-8 29673873 PMC6075697

[3] Adams SH Anthony JC Carvajal R Chae L Khoo CSH Latulippe ME. Perspective: guiding principles for the implementation of personalized nutrition approaches that benefit health and function. Adv Nutr. (2020) 11:25–34. 10.1093/advances/nmz086 31504115 PMC7442375

[4] Rozga M Latulippe ME Steiber A. Advancements in personalized nutrition technologies: guiding principles for registered dietitian nutritionists. J Acad Nutr Diet. (2020) 120:1074–85. 10.1016/j.jand.2020.01.020 32299678

[5] Celis-Morales C Lara J Mathers JC. Personalising nutritional guidance for more effective behaviour change. Proc Nutr Soc. (2015) 74:130–8. 10.1017/S0029665114001633 25497396

[6] Celis-Morales C Livingstone KM Marsaux CF Macready AL Fallaize R O'Donovan CB. Effect of personalized nutrition on health-related behaviour change: evidence from the Food4Me European randomized controlled trial. Int J Epidemiol. (2017) 46:578–88. 10.1093/ije/dyw186 27524815

[7] Hurkmans E Matthys C Bogaerts A Scheys L Devloo K Seghers J. Face-to-face versus mobile versus blended weight loss program: randomized clinical trial. JMIR Mhealth Uhealth. (2018) 6:e14. 10.2196/mhealth.7713 29326093 PMC5785684

[8] Eldredge L Markham C Ruiter R Fernandez M Kok G Parcel G. Planning Health Promotion Programs. An Intervention Mapping Approach. 4th ed. San Fransisco, CA: Jossey-Bass A Wiley Brand (2016).

[9] Vaitkeviciute R Ball LE Harris N. The relationship between food literacy and dietary intake in adolescents: a systematic review. Public Health Nutr. (2015) 18:649–58. 10.1017/S1368980014000962 24844778 PMC10271388

[10] Truman E Lane D Elliott C. Defining food literacy: a scoping review. Appetite. (2017) 116:365–71. 10.1016/j.appet.2017.05.007 28487244

[11] Vidgen HA Gallegos D. Defining food literacy and its components. Appetite. (2014) 76:50–9. 10.1016/j.appet.2014.01.010 24462490

[12] Amouzandeh C Fingland D Vidgen HA. A Scoping review of the validity, reliability and conceptual alignment of food literacy measures for adults. Nutrients. (2019) 11:801. 10.3390/nu11040801 30965628 PMC6520792

[13] Begley A Paynter E Dhaliwal SS. Evaluation tool development for food literacy programs. Nutrients. (2018)10:1617. 10.3390/nu10111617 30400130 PMC6267114

[14] Poelman MP Dijkstra SC Sponselee H Kamphuis CBM Battjes-Fries MCE Gillebaart M. Towards the measurement of food literacy with respect to healthy eating: the development and validation of the self perceived food literacy scale among an adult sample in the Netherlands. Int J Behav Nutr Phys Act. (2018) 15:54. 10.1186/s12966-018-0687-z 29914503 PMC6006995

[15] Grea Krause C Beer-Borst S Sommerhalder K Hayoz S Abel T. A short food literacy questionnaire (SFLQ) for adults: findings from a Swiss validation study. Appetite. (2017) 120:275–80. 10.1016/j.appet.2017.08.039 28912107

[16] Palumbo R Annarumma C Adinolfi P Vezzosi S Troiano E Catinello G. Crafting and applying a tool to assess food literacy: findings from a pilot study. Trends Food Sci Technol. (2017) 67:173–82. 10.1016/j.tifs.2017.07.002

[17] Nutbeam D. The evolving concept of health literacy. Soc Sci Med. (2008) 67:2072–8. 10.1016/j.socscimed.2008.09.050 18952344

[18] Sorensen K Van den Broucke S Fullam J Doyle G Pelikan J Slonska Z. Health literacy and public health: a systematic review and integration of definitions and models. BMC Public Health. (2012) 12:80. 10.1186/1471-2458-12-80 22276600 PMC3292515

[19] Sorensen K Van den Broucke S Pelikan JM Fullam J Doyle G Slonska Z. Measuring health literacy in populations: illuminating the design and development process of the European Health Literacy Survey Questionnaire (HLS-EU-Q). BMC Public Health. (2013) 13:948. 10.1186/1471-2458-13-948 24112855 PMC4016258

[20] Thomas H Azevedo Perry E Slack J Samra HR Manowiec E Petermann L. Complexities in conceptualizing and measuring food literacy. J Acad Nutr Diet. (2019) 119:563–73. 10.1016/j.jand.2018.10.015 30670348

[21] Boedt T Dancet E Lie Fong S Peeraer K De Neubourg D Pelckmans S. Effectiveness of a mobile preconception lifestyle programme in couples undergoing in vitro fertilisation (IVF): the protocol for the PreLiFe randomised controlled trial (PreLiFe-RCT). BMJ Open. (2019) 9:e029665. 10.1136/bmjopen-2019-029665 31366659 PMC6678004

[22] Boedt T Lie Fong S De Neubourg D Vereeck S Seghers J Van der Gucht K. Systematic development of a mobile preconception lifestyle programme for couples undergoing IVF: the PreLiFe-programme. Hum Reprod. (2021) 36:2493–505. 10.1093/humrep/deab166 34379119

[23] Polit DF Beck C. Nursing Research Principles and Methods. 4th ed. Philadelphia, PA: Lippincott Williams & Wilkins (2004).

[24] Patrick DL Burke LB Gwaltney CJ Leidy NK Martin ML Molsen E. Content validity–establishing and reporting the evidence in newly developed patient-reported outcomes (PRO) instruments for medical product evaluation: ISPOR PRO Good Research Practices Task Force report: part 2–assessing respondent understanding. Value Health. (2011) 14:978–88. 10.1016/j.jval.2011.06.013 22152166

[25] Patrick DL Burke LB Gwaltney CJ Leidy NK Martin ML Molsen E. Content validity–establishing and reporting the evidence in newly developed patient-reported outcomes (PRO) instruments for medical product evaluation: ISPOR PRO good research practices task force report: part 1–eliciting concepts for a new PRO instrument. Value Health. (2011) 14:967–77. 10.1016/j.jval.2011.06.014 22152165

[26] Lachat C Hawwash D Ocké MC Berg C Forsum E Hörnell A. Strengthening the reporting of observational studies in epidemiology-nutritional epidemiology (STROBE-nut): an extension of the STROBE statement. PLoS Med. (2016) 13:e1002036. 10.1371/journal.pmed.1002036 27270749 PMC4896435

[27] HogeGezondheidsraad. Voedingsaanbevelingen voor de Belgische volwassen bevolking met een focus op voedingsmiddelen. (2019).

[28] GezondLeven. https://www.gezondleven.be/ (2018). (accessed July 1, 2021).

[29] Broekhuis M van Velsen L Hermens H. Assessing usability of eHealth technology: a comparison of usability benchmarking instruments. Int J Med Inform. (2019) 128:24–31. 10.1016/j.ijmedinf.2019.05.001 31160008

[30] Qualtrics [Internet]. (2005) (first release). (Utah) Available online at: https://www.qualtrics.com

[31] Willett W. Nutritional Epidemiology. Oxford, UK: Oxford University Press (2013).

[32] Matthys C Meulemans A Schueren BVD. Development and validation of general FFQ for use in clinical practice. Ann Nutr Metab. (2015) 67:239.

[33] Huybrechts I Vereecken C De Bacquer D Vandevijvere S Van Oyen H Maes L. Reproducibility and validity of a diet quality index for children assessed using a FFQ. Br J Nutr. (2010) 104:135–44. 10.1017/S0007114510000231 20214836

[34] Wilson-Barlow L Hollins TR Clopton JR. Construction and validation of the healthy eating and weight self-efficacy (HEWSE) scale. Eat Behav. (2014) 15:490–2. 10.1016/j.eatbeh.2014.06.004 25064304

[35] Corp I IBM S. Statistics for Windows. Version 270. Armonk, NY: IBM Corp (2020).

[36] Azevedo Perry E Thomas H Samra HR Edmonstone S Davidson L Faulkner A. Identifying attributes of food literacy: a scoping review. Public Health Nutr. (2017) 20:2406–15. 10.1017/S1368980017001276 28653598 PMC10261432

[37] Truman E Bischoff M Elliott C. Which literacy for health promotion: health, food, nutrition or media? Health Promot Int. (2020) 35:432–44. 10.1093/heapro/daz007 30793740

[38] Kliemann N Beeken RJ Wardle J Johnson F. Development and validation of the self-regulation of eating behaviour questionnaire for adults. Int J Behav Nutr Phys Act. (2016) 13:87. 10.1186/s12966-016-0414-6 27484457 PMC4969721

[39] Kliemann N Wardle J Johnson F Croker H. Reliability and validity of a revised version of the general nutrition knowledge questionnaire. Eur J Clin Nutr. (2016) 70:1174–80. 10.1038/ejcn.2016.87 27245211 PMC5014128

[40] Ridder KD. Samenvatting van de resultaten. Voedselconsumptiepeiling 2014–2015. WIV-ISP, Brussel. In: Bel S BL, Lebacq T, Ost C & Teppers E, editors (2016).

[41] Djojosoeparto SK Kamphuis C Vandevijvere S Poelman MP. The Healthy Food Environment Policy Index (Food-EPI): Nederland. Een beoordeling van rijksoverheidsbeleid met betrekking tot de voedselomgeving in Nederland en beleidsaanbevelingen voor het creëren van een gezonde voedselomgeving Universiteit Utrecht (2020).

[42] Boateng GO Neilands TB Frongillo EA Melgar-Quiñonez HR Young SL. Best practices for developing and validating scales for health, social, and behavioral research: a primer. Front Public Health. (2018) 6:149. 10.3389/fpubh.2018.00149 29942800 PMC6004510

[43] Hebert JR Clemow L Pbert L Ockene IS Ockene JK. Social desirability bias in dietary self-report may compromise the validity of dietary intake measures. Int J Epidemiol. (1995) 24:389–98. 10.1093/ije/24.2.389 7635601

[44] Murakami K Shinozaki N Masayasu S Livingstone MBE. Web-based personalized nutrition system for delivering dietary feedback based on behavior change techniques: development and pilot study among dietitians. Nutrients. (2021) 13:3391. 10.3390/nu13103391 34684392 PMC8538565

