# Supplementary material for: A Mixed-Method Approach to Develop and Validate an Integrated Food Literacy Tool for Personalized Food Literacy Guidance
Source: Front Nutr. 2022 Jan 21;8:760493. doi: 10.3389/fnut.2021.760493 (PMC8814651; doi:10.3389/fnut.2021.760493)

A) Distribution of total food literacy score among men and women

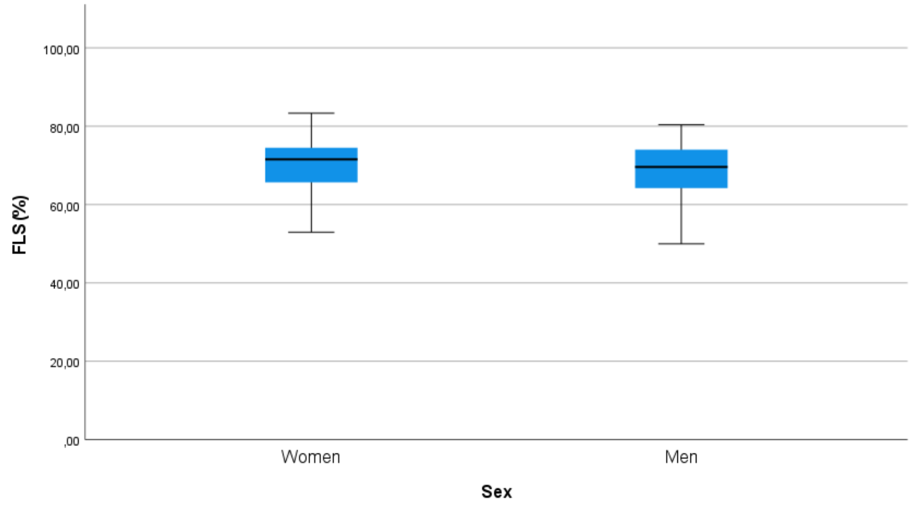

B) Distribution of total food literacy score among people with or without a childwish

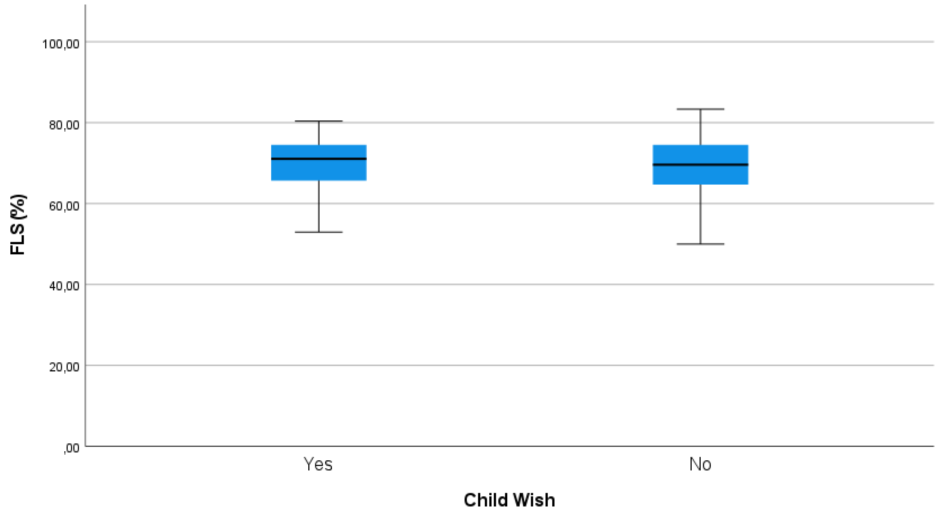

C) Correlation between total food literacy score and age

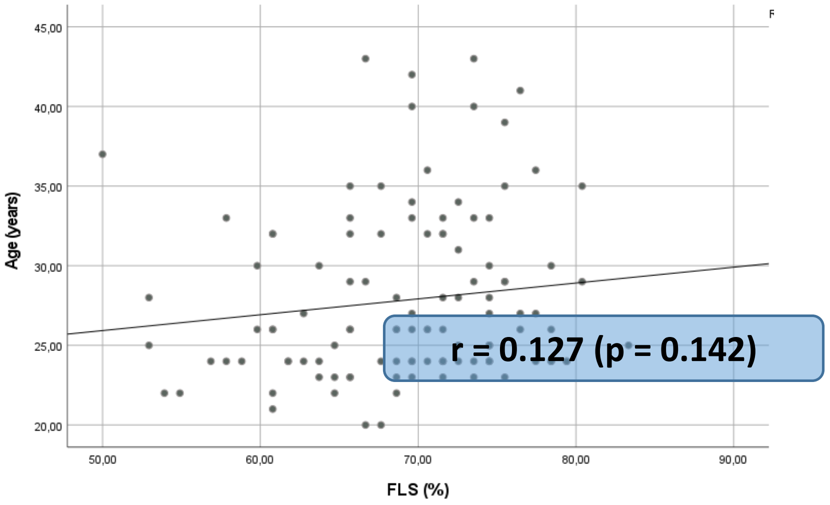

D) Correlation between total food literacy score and education level

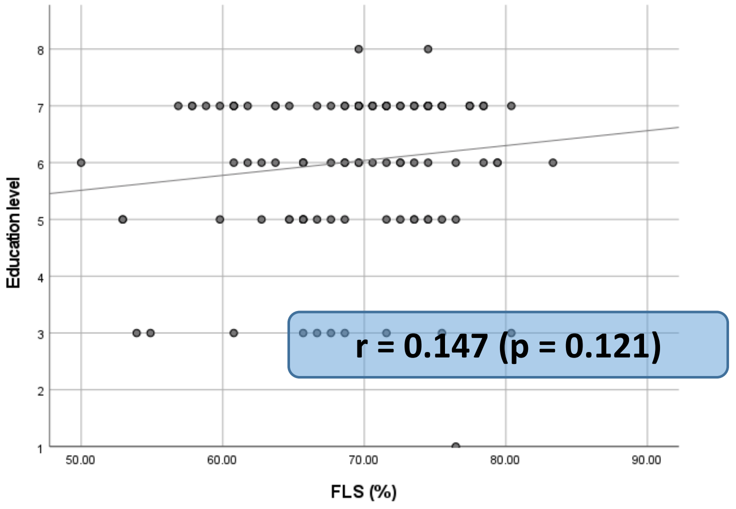

E) Correlation between total food literacy score and self-reported health

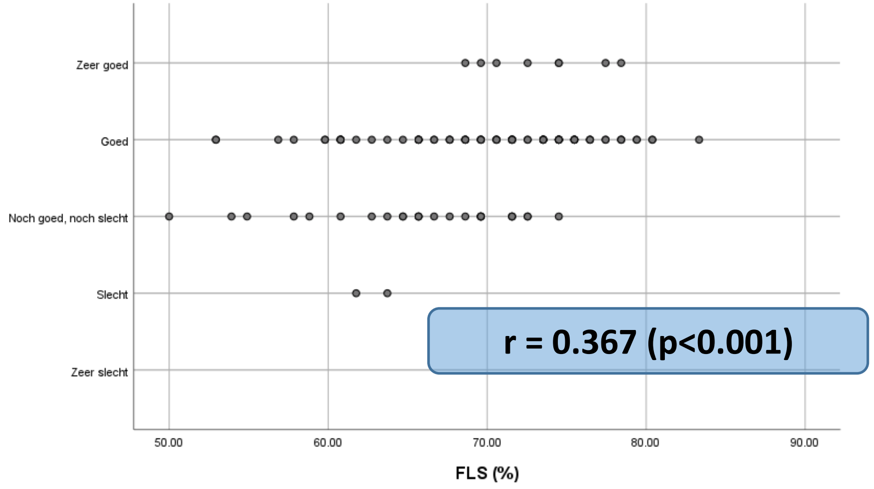

Supplement: Supplementary file 1 [file Image_1.pdf]
